# Structural brain differences in pre-adolescents who persist in and recover from stuttering

**DOI:** 10.1016/j.nicl.2020.102334

**Published:** 2020-06-29

**Authors:** S.P.C. Koenraads, M.P. van der Schroeff, G. van Ingen, S. Lamballais, H. Tiemeier, R.J. Baatenburg de Jong, T. White, M.C. Franken, R.L. Muetzel

**Affiliations:** a Department of Otorhinolaryngology and Head and Neck Surgery, Erasmus University Medical Center, Rotterdam, the Netherlands; b The Generation R Study Group, Erasmus University Medical Center, Rotterdam, the Netherlands; c Department of Epidemiology, Erasmus University Medical Center, Rotterdam, the Netherlands; d Department of Clinical Genetics, Erasmus University Medical Center, Rotterdam, the Netherlands; e Department of Child and Adolescent Psychiatry/Psychology, Erasmus University Medical Center, Rotterdam, the Netherlands; f Department of Social and Behavioral Sciences, Harvard T.H. Chan School of Public Health, Boston, MA, United States

**Keywords:** CBCL, child behavior checklist, DTI, diffusion tensor imaging, FA, fractional anisotropy, FAT, frontal aslant tract, FDR, false discovery rate, MD, mean diffusivity, MRI, magnetic resonance imaging, ICV, intracranial brain volume, IFG, inferior frontal gyrus, IQ, intelligence quotient, Stuttering, Brain development, Childhood-onset fluency disorder, Pre-adolescents

## Abstract

**Background:**

Stuttering is a complex speech fluency disorder occurring in childhood. In young children, stuttering has been associated with speech-related auditory and motor areas of the brain. During transition into adolescence, the majority of children who stutter (75–80%) will experience remission of their symptoms. The current study evaluated brain (micro-)structural differences between pre-adolescents who persisted in stuttering, those who recovered, and fluently speaking controls.

**Methods:**

This study was embedded in the Generation R Study, a population-based cohort in the Netherlands of children followed from pregnancy onwards. Neuroimaging was performed in 2211 children (mean age: 10 years, range 8–12), of whom 20 persisted in and 77 recovered from stuttering. Brain structure (e.g., gray matter) and microstructure (e.g., diffusion tensor imaging) differences between groups were tested using multiple linear regression.

**Results:**

Pre-adolescents who persisted in stuttering had marginally lower left superior frontal gray matter volume compared to those with no history of stuttering (β −1344, 95%CI −2407;-280), and those who recovered (β −1825, 95%CI −2999;-650). Pre-adolescents who recovered, compared to those with no history of stuttering, had higher mean diffusivity in the forceps major (β 0.002, 95%CI 0.001;0.004), bilateral superior longitudinal fasciculi (β 0.001, 95%CI 0.000;0.001), left corticospinal tract (β 0.003, 95%CI 0.002;0.004), and right inferior longitudinal fasciculus (β 0.001, 95%CI 0.000;0.001).

**Conclusion:**

Findings suggest that relatively small difference in prefrontal gray matter volume is associated with persistent stuttering, and alterations in white matter tracts are apparent in individuals who recovered. The findings further strengthen the potential relevance of brain (micro-)structure in persistence and recovery from stuttering in pre-adolescents.

## Highlights

• Brain (micro-)structural differences were found in pre-adolescents who stutter.
• Persistency was associated with marginally smaller left frontal gray matter volume.
• Recovery was associated with higher mean diffusivity in white matter tracts.
• Distinct brain structures implicated in persistence and recovery of stuttering.

## 1 Introduction

Childhood-onset fluency disorder, commonly referred to as stuttering, is a complex developmental speech production disorder that occurs in childhood between two and five years of age (American Psychiatric Association, 2013, Yairi and Ambrose, 1999). Stuttering occurs in 5–10% of preschool-age children (Boyle et al., 2011, Craig et al., 2002, Dworzynski et al., 2007, Månsson, 2000, Reilly et al., 2013). Most children who stutter recover spontaneously (75–80%) within 2–3 years after onset (Andrews and Harris, 1964, Kefalianos et al., 2017, Månsson, 2000, Reilly et al., 2013, Rommel et al., 1999, Yairi and Ambrose, 2013). Persistent stuttering impacts social and professional communication in adults, as well as overall quality of life (Beilby et al., 2013, Craig et al., 2009, Finn, 2003, Klompas and Ross, 2004, Koedoot et al., 2011, Nang et al., 2018, Yaruss and Quesal, 2006). Current views on the etiology of developmental stuttering are multifactorial, combining genetic, neurobiological, psychological and environmental factors (Smith & Weber, 2017). Identifying the brain structures involved in stuttering and the accompanying developmental course may lead to new opportunities for neuroscience-informed therapies.

There is increasing evidence for differences in gray matter structure (Beal et al., 2013, Chang et al., 2008, Foundas et al., 2013, Garnett et al., 2018, Koenraads et al., 2019) and white matter micro-structure (Beal et al., 2013, Chang et al., 2015, Chow and Chang, 2017) in the brains of children who stutter compared to their fluently speaking peers. Neuroimaging studies in adults whose stuttering persisted have also shown differences in brain (micro-)structure (Etchell, Civier, Ballard, & Sowman, 2018). The most common finding across morphometric studies in children and adults who stutter are differences in the left hemisphere speech network. In cortical gray matter structures, specifically, the pars- opercularis, triangularis and orbitalis, smaller volume and thinner cortex were reported in children who stutter compared to fluent peers (Beal et al., 2013, Garnett et al., 2018, Koenraads et al., 2019). Smaller gray matter volume and thinner cortex in the right frontal lobe have also been previously reported (Beal et al., 2013, Koenraads et al., 2019). In subcortical structures, smaller volumes in the basal ganglia were observed in children who stutter. The left putamen and right caudate have also been associated with stuttering, both of which play an important role in smooth speech motor control and adequate timing and rhythm (Beal et al., 2013, Bohland and Guenther, 2006, Foundas et al., 2013, Grahn, 2012). Further, in adults with persistent stuttering, smaller gray matter volumes were shown in regions involved in the left inferior frontal gyrus (IFG) as well as the bilateral precentral and postcentral gyri, compared fluent controls (Kell et al., 2009, Lu et al., 2010). In contrast, larger gray matter volume in similar regions have also been reported in adults who stutter (Beal et al., 2007, Lu et al., 2010, Song et al., 2007).

White matter microstructural metrics also differed in children who stutter compared to fluent peers along the left arcuate fasciculus, left superior longitudinal fasciculus (SLF), bilateral corticospinal tracts, and in the interhemispheric corpus callosum fibers (Chang et al., 2008, Chow and Chang, 2017). Chow and Chang (2017) found that children who recover from stuttering could be differentiated from those who persist by distinct neurodevelopmental trajectories; the recovered group showed normalized white matter growth with age, and the persistent group showed a reduction of growth rate. Similarly, in adults with persistent stuttering compared to fluent controls, lower fractional anisotropy (FA) was also reported in the left perisylvian region, left SLF, forceps minor, and body of the corpus callosum (Cykowski et al., 2010, Sommer et al., 2002, Watkins et al., 2008). Studies including both children and adults that identified brain areas with white matter microstructural properties, found lower FA in stuttering than in fluent speakers. The discrepancy in results among studies might reflect sampling differences in participants, including age differences, statistical significance thresholds (Cykowski et al., 2010, Sommer et al., 2002), or perhaps heterogeneity across individuals in the spatial location of affected brain areas.

Although (micro-)structural brain regions associated with auditory function and motor aspects of speech have been associated with stuttering, several gaps exist in the literature. First, with the exception of studies by Chang and colleagues investigating brain morphometry in (pre-)adolescents (ages 9–12 years), the examination of recovery from and persistence in stuttering has been limited (Chang et al., 2008, Chow and Chang, 2017, Garnett et al., 2018). Understanding the neurobiological determinants or consequences of recovery will be a crucial step towards personalized and targeted therapies in the future. Second, the vast majority of work focuses on case-control designs which is potentially biased by a.) recruitment of severely affected individuals who stutter and b.) reference samples (control groups) which are substantially different from cases in terms of, for example, certain demographic characteristics. Sampling cases and the reference sample from the general population allows for more variability in the distribution in the case group (i.e., not only the most severely affected children are included) and a reference group which is better sampled on several factors (e.g., SES), thereby likely improving generalizability. Given this background, studying brain morphometry in relation to stuttering in pre-adolescents is essential to further increase our knowledge of the neural architecture of persistency in and recovery from stuttering.

This study aimed to build upon earlier work (Koenraads et al., 2019) and explored whether there were gray and white matter differences in a large population-based cohort of pre-adolescents ages 8-to-12 years amongst those who persisted in stuttering, those who recovered, and fluently speaking peers. Based on prior findings, we hypothesized that brain regions involved in speech production will show differences (e.g., smaller volume, thinner cortex, lower FA) in pre-adolescents who persisted in stuttering compared with those who had recovered and controls who have always spoken fluently. We further hypothesized these differences to be primarily concentrated in the cortical auditory-motor network of the left hemisphere, in the subcortical basal ganglia nuclei, and in white matter underlying those auditory-motor network areas. Finally, we hypothesized that, compared to those who never stuttered, differences in brain regions underlying stuttering in pre-adolescents who persist in stuttering will be larger than differences observed in the group of children who recovered from stuttering.

## 2 Methods

### 2.1 Study design and population

**Fig. 1:**
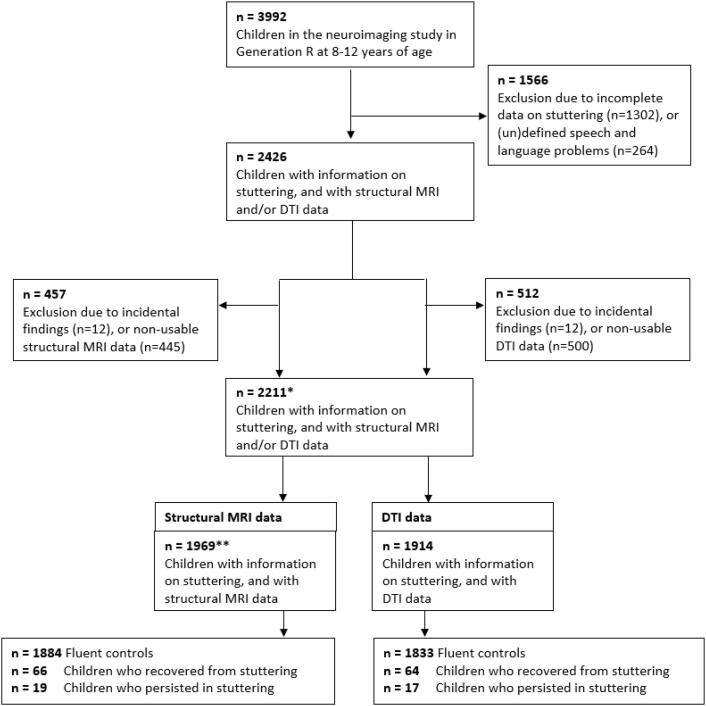
Flowchart of sample selection. * this number of participants is used in Table 1 for participant demographics; ** this number of participants is used in Supplementary Table 1 for participant demographics. DTI = Diffusion Tensor Imaging, MRI = Magnetic Resonance Imaging, n = number.

This study was embedded in the Generation R Study, a multi-ethnic population-based prospective cohort from fetal life onward in Rotterdam, the Netherlands (Kooijman et al., 2016). The Generation R Study follows children of mothers who were living in Rotterdam with a delivery date from April 2002 to January 2006. Parents of all participating children provided written informed consent and the study was approved by the Medical Ethics Committee of Erasmus Medical Center. Children underwent brain MRI scans when they were ten years old starting in March 2013 (White et al., 2018). Neuroimaging data was available for 3992 children (Fig. 1). Children with incomplete information on stuttering or other (un)defined speech and language problems (e.g., based on speech and language history parental questionnaire) were excluded (n = 1566). Additionally, children with poor MR image quality (n = 445 in structural MRI (T_1_-weighted), n = 500 in DTI) as well as children with major incidental findings (n = 12) were excluded from analyses. In total, 1969 datasets were available for T_1_-weighted MRI analysis and n = 1914 for DTI analysis).

### 2.2 Stuttering

When the children were around nine years old, their parents completed a speech and language developmental questionnaire to obtain a measure of childhood stuttering (Koenraads et al., 2019). The questionnaire contained two questions specifically regarding stuttering and had categorical items: “Does your child currently stutter?” and “Has your child ever stuttered in the past?” (yes/no). Two questions about speech therapy were assessed as well: “Is your child currently being treated for stuttering?” and “Has your child ever been treated for stuttering in the past?” (yes/no). Participants were classified as children who persisted in stuttering if both questionnaires, about the past and the present, were endorsed. Similarly, participants were classified as having recovered from stuttering if parents only endorsed previous stuttering and not current stuttering. Based on parent report, treatment for stuttering was retrospectively categorized as either: ever had treatment for stuttering, or ever had treatment for stuttering, speech and/or language problems. This categorization was based on parental reports only, and has been used in several previous studies investigating persistency in and recovery from stuttering in childhood (Dworzynski et al., 2007, Kloth et al., 1999, Koenraads et al., 2019, Månsson, 2000). Parents of children who stutter and parents of fluently speaking children can accurately and reliably identify stuttering (Bloodstein and Bernstein Ratner, 2008, Einarsdottir and Ingham, 2009).

### 2.3 Magnetic resonance imaging

An overview of the imaging procedure, acquisition, sequences, and quality assessment in the Generation R neuroimaging Study has been described previously (White et al., 2018). All children were familiarized with the scanning environment in a mock scanning session prior to the actual MRI scanning session. Brain images were acquired on a 3 Tesla scanner (General Electric Discovery MR750w, Milwaukee, WI, USA) with an eight-channel head coil. High-resolution T_1_-weighted images were obtained with a 3D inversion recovery fast-spoiled gradient recalled sequence (TR = 8.77 ms, TE = 3.4 ms, TI = 600 ms, Flip Angle = 10°, Field of View (FOV) = 220x220 mm, Acquisition Matrix = 220x220, slice thickness = 1 mm, number of slices = 230, Parallel Imaging Factor (ARC) = 2). The diffusion weighted images were collected with an axial spin echo, echo-planar imaging sequence with three volumes with b = 0 s/mm^2^ and 35 diffusion-weighted images (TR = 12,500 ms, TE = 72.8 ms, FOV = 240x240 mm, Acquisition Matrix = 120x120, slice thickness = 2 mm, number of slices = 65, Parallel Imaging Factor (ASSET) = 2, b = 900 s/mm^2^).

### 2.4 Image processing

Cortical reconstruction and volumetric segmentation were conducted with the FreeSurfer image suite version 6.0 (http://surfer.nmr.mgh.harvard.edu/) (Fischl, 2012). To summarize, removal of non-brain tissue (e.g., skull strip), voxel intensity normalization, initial tissue segmentation, cortical reconstruction and automated anatomical labeling were performed. The anatomical metrics we measured for this study were: gray matter volumes for subcortical and cortical structures, white matter volume of the corpus callosum and surface-based thickness and surface area of the cortex.

The diffusion tensor imaging (DTI) data was processed with the FMRIB Software Library (FSL) (Jenkinson, Beckmann, Behrens, Woolrich, & Smith, 2012), and the Camino diffusion MRI toolkit (Cook et al., 2006). Non-brain tissue was removed and images were corrected for motion and eddy-current artifacts. The resulting transformation matrices were used to rotate the gradient direction table to account for rotations applied to the data. The diffusion tensor was fitted at each voxel with FSL (BEDPOSTx package) (https://fsl.fmrib.ox.ac.uk/fsl/fslwiki/FDT/UserGuide#BEDPOSTX), and the most common scalar metrics (e.g., FA and mean diffusivity (MD)) were computed. The full procedure is described previously (Muetzel et al., 2018). Fully-automated probabilistic tractography was performed to estimate connectivity distributions for a number of large fiber bundles using the FSL Probtrackx module with a set of predefined seed and target masks supplied by the FSL plugin, AutoPtx (https://fsl.fmrib.ox.ac.uk/fsl/fslwiki/AutoPtx) (de Groot et al., 2015). The number of samples passing through a given voxel on a successful seed-to-target run were registered, and the resulting distributions were normalized (by the number of total successful seed-to-target attempts) and low-probability voxels were removed. In order to estimate a ‘global’ indicator of FA and MD, analogous to total brain volume in structural MRI, mean FA and MD were extracted from each tract, and confirmatory factor analysis was used to generate latent FA and MD measures across 12 tracts (forceps major and minor, and the left and right cingulum bundle, corticospinal tract, inferior and superior longitudinal fasciculus (ILF, SLF), and the uncinate fasciculus) which represent global white matter microstructure across the brain (Muetzel et al., 2018). The confirmatory factor analysis determines a linear combination of all tracts in order to estimate a single factor which maximally explains the variability of these tracts. This measure from now on is referred to as global FA and global MD.

FreeSurfer image reconstructions of the T1-weighted images were visually inspected for quality. All scans rated as unusable were excluded from statistical analyses (Muetzel et al., 2019). Diffusion image quality was assessed by manual inspection (visualization of residual error maps from the tensor fit and inter-subject registration) and automated methods (DTIprep toolkit, https://www.nitrc.org/projects/dtiprep) (Muetzel et al., 2018).

### 2.5 Covariates

In order to minimize the effect of confounding bias, all analyses were adjusted for several covariates. Child sex and age were calculated based on birth records. Child ethnicity was defined according to the classification of the Statistics Netherlands institute, into Western (i.e., Dutch and other non-Dutch Western) and Non-Western (Statistics Netherlands, 2004). Mono- or bilingualism was defined as acquiring one or more than one native language by age of six years through their parents in the home environment, and was assessed via questionnaires (Peng & Wang, 2011). Handedness (tendency to be right or left-handed) was determined using the Edinburg Handedness Inventory (Oldfield, 1971). Child non-verbal intelligence quotient (IQ) was assessed in the research center when children were five to seven years old, with a validated Dutch non-verbal intelligence test: Snijders-Oomen Niet-verbale intelligentie test, 2.5–7- revisie (SON-R 2.5–7) (Tellegen, Winkel, Wijnberg-Williams, & Laros, 2005). A total behavioral and emotional problem score was quantified using parental reported Child Behavior Checklist (CBCL for ages 6–18) when children were approximately nine years old. The CBCL is a widely used reliable and valid instrument that measures behavioral and emotional problems in children (Achenbach and Rescorla, 2000, Achenbach and Ruffle, 2000).

Maternal educational level was categorized following the definitions of Statistics Netherlands into two categories: medium or low (none, high school or some vocational training), and high educational attainment (higher vocational education or university) (Statistics Netherlands., 2005).

### 2.6 Data analysis

For main analyses, we divided the sample into three groups: children whose stuttering persisted, children who recovered from stuttering, and fluently speaking controls. We examined associations of the groups with brain morphology and white matter microstructure. First, the reference sample was compared with both children who persisted in their stuttering over time, and those who recovered from stuttering over time. Second, in analyses where differences were observed with the reference sample, we also compared brain metrics of children who persisted in stuttering to those who recovered in order to ascertain whether those who persisted showed larger differences than those who recovered. Differences in demographic variables between the different stuttering groups were determined using Chi-squared and Fisher’s exact tests for categorical variables and t-tests for continuous variables.

The *a priori* regions of interest in structural gray matter (i.e., cortical volume, thickness and surface area, and subcortical volume) and white matter (i.e., volume) were our primary outcomes. Specifically, based on prior literature on gray matter, we selected the left and right frontal and temporal lobe, the perisylvian frontotemporal regions (e.g., IFG, insula), the premotor and primary motor regions (e.g., post- and precentral gyrus), and the parietal lobe with supramarginal gyrus (Chang et al., 2008, Garnett et al., 2018, Koenraads et al., 2019). We also tested whether stuttering was associated with total brain volume. Additionally, we selected the following subcortical structures: basal ganglia (caudate, putamen, pallidum) and thalamus. Further, for white matter structures, we chose to examine the five main components of the corpus callosum volume quantified in FreeSurfer (5 mm thick midline section) separately (Beal et al., 2013). Our second set of outcomes were measures of white matter microstructure (FA and MD), derived from DTI. Specifically, we selected 14 different fiber bundles, including the forceps minor and major, and the left and right ILF and SLF, uncinate fasciculus, inferior fronto-occipital, corticospinal tract, and the posterior thalamic radiations (Chow & Chang, 2017). As different terms such as microstructure and integrity have been used to describe the constructs measured by DTI (Jones, Knosche, & Turner, 2013), we would like to clarify on the terminology used in this paper. We refer to DTI metrics as describing white matter microstructure, consistent with a substantial proportion of the DTI literature. However, confusion can arise in this terminology, as the resolution of DTI data are typically such that many hundreds of thousands of neurons are sampled within a single voxel, which is far from the micro-scale. The tissue sampled in this study is certainly at the macroscale, however, the signal underlying the diffusion metrics is the result of various microscale physiological processes.

We examined the association between stuttering groups and brain outcomes with multiple linear regression models. We adjusted models for the following confounders: child sex, age, handedness, bilingualism, ethnicity, and maternal education. We further added intracranial brain volume (ICV) in models with volumetric outcomes. For all brain structural measures, regression beta coefficients (β) are presented the of millimeter scale (e.g., volume in mm^3^, surface area in mm^2^, thickness in mm, FA unitless with a value between 0 and 1, MD have been scaled by a factor of 1000 and are reported in 10^3^ mm2/sec).

Non-response analyses were conducted, contrasting the group of children who had missing data (n = 1566), or non-usable data (n = 215) to our study sample. Several supplemental analyses were also conducted. First, given the large number of covariates, we reran models only adjusting for age and sex in order to rule out overfitting. Second, given the complex relationship between stuttering and child’s cognition and behavior, we adjusted our analyses in separate models for child’s intelligence score and for total behavior problems to test the specificity of the effect. Third, analyses were performed in boys only, since stuttering is more common in boys, and to ascertain the robustness of findings without girls.

All analyses were performed in SPSS (version 24.0) and R statistical software (version 3.5.1). Missing values of covariates (maximum percentage: bilingualism = 6.1%) were replaced by using multiple imputation to generate five data sets that sampled these values from their predictive distribution based on the relations between all variables included in the present study (Sterne et al., 2009). Results were pooled according to standard procedures (Rubin, 2004). False Discovery Rate (FDR) correction was applied to correct for the number of tests (Benjamini & Hochberg, 1995). Correction was applied separately for morphological (e.g., volume) and DTI (e.g., FA).

## 3 Results

### 3.1 Description of study sample

Summarized in Table 1, 20 children persisted in stuttering and 77 recovered from stuttering, which is 97 of the 2211 children (4%) were classified as having a history of stuttering by nine years old (SD = 0.3 year). Boys were more likely to persist in stuttering than girls (80% boys, p-value < 0.05). Children in this study were mostly of Western ethnicity (77%), were monolingual (80%), and were right handed (89%). Seventy-five percent of children in the persistent group ever had treatment for stuttering, and 39% in the recovered group. Non-response analyses showed that children who participate in the current study were more likely to have Western ethnicity, and higher educated mothers than children who did not participate in the study. Demographics for all participants with T_1_-weighted data are summarized in Supplementary Table A.1.

**Table 1:** Demographics for all participants with structural and/or DTI MRI data (n = 2211).

	Fluent controls n = 2114	Children who recovered from stuttering n = 77	Children who persisted in stuttering n = 20	p-value
Child				
Age at questionnaire (years)	9.8 (0.3)	9.8 (0.2)	9.8 (0.4)	> 0.05
Age at MRI (years)	10.1 (0.6)	10.0 (0.5)	9.9 (0.6)	> 0.05
Sex (% boy)	1018 (48.2)	53 (68.8)	16 (80.0)	0.01^a^, 4E-4^b^
Ethnicity (% Western)	1632 (77.7) [n = 2100]	54 (71.1)	13 (65.0)	> 0.05
Handedness (% right)	1883 (89.4) [n = 2106]	70 (92.1) [n = 76]	16 (80.0)	> 0.05
Lingualism (% monolingual)	1667 (80.3) [n = 2075]	60 (77.9)	15 (70.0)	> 0.05
Ever had stutter therapy (% yes)	-	29 (37.7)	15 (75.0)	0.01*
Ever had stutter, speech and/or language therapy (%yes)	317 (17.5)	34 (44.2)	16 (80.0)	2E-9^a^, 1E-7^b^, 0.01^c^
IQ (score)	104.9 (14.3) [n = 1871]	102.6 (15.8) [n = 67]	103.8 (15.1) [n = 18]	> 0.05
Total behavior behavior score (mean, SD)	15.9 (14.1) [n = 2024]	16.3 (13.6) [n = 76]	25.6 (18.6) [n = 19]	3E-3^a^^,^ 0.01^c^
Total problem behavior score (median, IQR)	12.0 (6.0 – 22.0)	13.5 (7.0 – 20.0)	23.0 (10.1 – 36.3)	4E-3^a^^,^ 0.01^c^
Maternal				
Ethnicity (%Western)	1566 (74.6) [n = 2098]	54 (71.1) [n = 76]	12 (60.0)	> 0.05
Education level (% high)	1332 (67.1) [n = 1984]	51 (68.9) [n = 74]	8 (47.1) [n = 17]	> 0.05

All categorical variables are presented with numbers (n) and percentages (%); all continuous variables are presented as mean with standard deviation (SD).

IQ = intelligence quotient, MRI = magnetic resonance imaging, n = number

a persistent stuttering > fluent controls (p-value < 0.05)

b recovered stuttering > fluent controls (p-value < 0.05)

c persistent stuttering > recovered stuttering (p-value < 0.05)

### 3.2 Gray matter morphometry

**Fig. 2:**
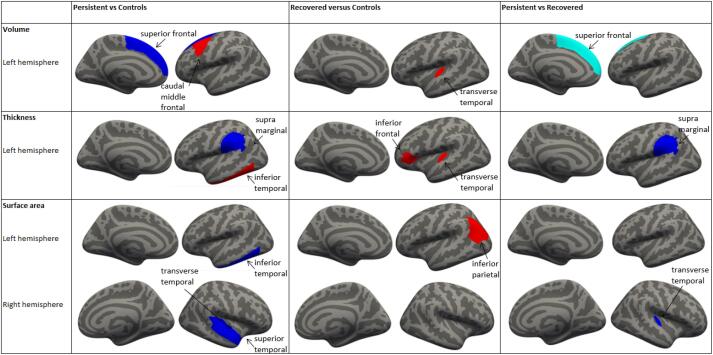
Gray matter morphometry differences associated with stuttering, red = positive, did not survive FDR; yellow = positive, pFDR < 0.05; dark blue = negative, did not survive FDR; light blue = negative, pFDR < 0.05.

Stuttering was associated with structural morphology in pre-adolescents. Fig. 2 demonstrates marginally smaller gray matter volume in the left superior frontal lobe in children who persisted in stuttering compared to those with no history of stuttering (β −1344, 95%CI −2407;-280, *p*_FDR_ = 0.16) and also versus those who recovered (β −1825 95%CI −2999;-650, *p*_FDR_ = 0.046). Note, this difference between children who persisted and those with no history of stuttering was non-significant after correction for multiple testing. Many other cortical brain regions of interest were also associated, but did not survive correction for multiple testing (Supplementary Table B.1, C.1 and D.1–2). No association was observed between stuttering and the global brain measure.

In the subcortical structures, the volumes of the basal ganglia and the thalamus were similar across all groups in both hemispheres (Supplementary Table B.1 and C.1, p_FDR_ > 0.05).

### 3.3 White matter volume and microstructure

Corpus callosum volumes were not associated with stuttering (Supplementary Table B.1 and C.1–2, p_FDR_ > 0.05).

**Fig. 3:**
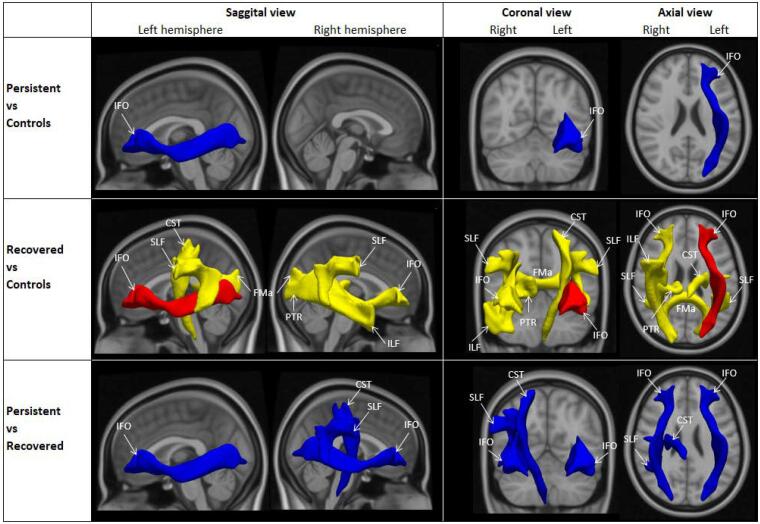
White matter tracts differences associated with stuttering, in mean diffusivity (MD). CST = corticospinal tract, FMa = forceps major, IFO = inferior fronto-occipital, ILF = inferior longitudinal fasciculus, SLF = superior longitudinal fasciculus, PTR = posterior thalamic radiation, red = positive, did not survive FDR; yellow = positive, pFDR < 0.05; dark blue = negative, did not survive FDR; light blue = negative, pFDR < 0.05.

DTI results showed that stuttering was associated with white matter microstructure in pre-adolescents (Fig. 3 and Table 2). An association was observed between stuttering and the global MD, and not between stuttering and global FA measure. Children who recovered from stuttering had higher MD in the forceps major (β 2.119, 95%CI 0.505;3.732, *p*_FDR_ = 0.02), bilateral SLF (left β 0.848, 95%CI 0.292;1.404, *p*_FDR_ = 0.01; right β 1.018, 95%CI 0.375;1.660, *p*_FDR_ = 2E-3), left corticospinal tract (β 3.175, 95%CI 1.854;4.495, *p*_FDR_ = 3E-5), right ILF (β 1.307, 95%CI 0.538;2.076, *p*_FDR_ = 0.01), right inferior frontal-occipital (β 0.783, 95%CI 0.164;1.401, *p*_FDR_ = 0.04), and right posterior thalamic radiation (β1.369, 95%CI 0.374;2.364, *p*_FDR_ = 0.02) compared to controls (Supplementary Table E.1). Other *a priori* white matter tracts showed significance before, but not after, adjusting for multiple comparisons (p_FDR_ > 0.05, in Fig. 3 and Table 2). Children who persisted in stuttering had similar MD compared to controls, and to those who had recovered. FA in pre-specified tracts were similar across all groups in both hemispheres (p_FDR_ > 0.05).

**Table 2:** Overview of DTI differences associated with stuttering, mean diffusivity (MD).

Regions#	Left hemisphere	Right hemisphere
Superior longitudinal fasciculus	R > C*	R > C*, P < R+
Inferior longitudinal fasciculus	-	R > C*
Forceps minor^o^	-	
Forceps major ^o^	R > C*	
Uncinate fasciculus	-	-
Inferior fronto-occipital	P < C+, R > C+, P < R+	R > C*, P < R^+^
Corticospinal tract	R > C*	P < R^+^
Posterior thalamic radiation	-	R > C*
	/	

P = persistent, R = recovered, C = fluent controls

Linear regression model: brain tract of interest = stuttering + age + gender + handedness + bilingualism + ethnicity + maternal education

The number of participants varied in each analysis: P vs R (n = 1850), P vs R (n = 81), R vs C (n = 1897).

# regions are selected based on previous literature

o forceps minor and major: one brain region of interest, not left and right separately

* p_FDR_ < 0.05

+ significant p-value < 0.05 and did not survive FDR correction (Chang et al., 2008, Foundas et al., 2013, Garnett et al., 2018, Koenraads et al., 2019).

### 3.4 Sensitivity analysis

In sensitivity analyses, results remained highly similar in the model when only adjusting for age and sex as covariates, suggesting overfitting is not an issue in the fully adjusted model. Second, the effect estimates (referring to standardized regression coefficients) of the association of stuttering with gray and white matter structures remained highly consistent when adjusting for IQ and behavioral problems, suggesting that these factors are not explaining the results. Third, brain (micro-)structural associations in analyses of boys only were similar to the original analyses with boys and girls, suggesting that results were not driven by the relatively small number of girls in the stuttering group.

## 4 Discussion

The goal of this study was to determine whether pre-adolescents who stutter show distinct neuroanatomical features when compared to peers who do not stutter and to those who recovered from stuttering. Results indicate that volume of the left superior frontal lobe is smaller with persistent stuttering, athough the effect size (standardized regression coefficient) is very small. Recovery was associated with higher MD in white matter microstructure interconnecting frontotemporal regions and sensorimotor areas of the cortex compared to fluently speaking peers. These results improve our insight into neural underpinnings of speech that are relevant for stuttering persistence and recovery in pre-adolescence.

After correcting for multiple testing, there were no significant differences in cortical gray matter structures in pre-adolescents with a history of stuttering compared to those with no history of stuttering. However, our data suggest the potential for some very subtle gray matter structural differences, for example smaller left superior frontal volume in children who persist in stuttering. This is consistent with earlier published studies in children with stuttering who reported smaller gray matter volume and thinner left frontotemporal cortex (Garnett et al., 2018, Koenraads et al., 2019) and with studies in adults with persistent stuttering who reported smaller gray matter volumes in the left IFG (Kell et al., 2009, Lu et al., 2010). Our subtle finding is in contrast with studies which reported smaller gray matter volume and thinner cortex in the right frontal lobe in children who stutter (Beal et al., 2013, Koenraads et al., 2019), or larger gray matter volume in left frontotemporal regions in adults who persist in stuttering (Beal et al., 2007, Song et al., 2007). Interestingly, the frontal lobes are known for neurodevelopmental differences that might contribute to the manifestation of stuttering in pre-adolescents (e.g., in the domain of emotions, behavior, motor skills, or language) (Blomgren, 2013, Smith and Weber, 2017). A potential mechanism that may underlie the smaller frontal gray matter findings is related to the general spatio-temporal development of brain macrostructure, where frontal areas mature later in adolescence (Giedd et al., 1996). Children with persistent stuttering may have a delay in growth in these frontal regions (e.g., slow maturation) (Thapar & Rutter, 2015). Those who spontaneously recover could experience a form of ‘catch up’ growth compared to those who persist. However, as these speculations were based on cross-sectional findings, as well as our findings, directionality between stuttering and brain development cannot be distinguished without longitudinal data. Further, the medial aspect of the frontal region is innervated by the left frontal aslant tract (FAT), which interconnects the left IFG with the pre-supplementary and supplementary motor area, and the cingulate gyrus, and is shown to support language production (Dick, Garic, Graziano, & Tremblay, 2019). More specific to stuttering, recent studies have found altered microstructure of the FAT in children and adults who stutter (Chow and Chang, 2017, Kronfeld-Duenias et al., 2016b, Misaghi et al., 2018, Neef et al., 2018), suggesting abnormal connectivity amongst these interconnected regions could explain the macrostructural frontal lobe differences.

Other a priori defined subcortical gray matter structures were not associated with stuttering after adjusting for multiple testing. This is in contrast with studies which found the subcortical left putamen and right caudate to be associated with stuttering in right-handed boys who stutter (Beal et al., 2013, Foundas et al., 2013). We did not find any related subcortical regions (e.g., those involved voluntary movement) to stuttering in our previous study of young children (Koenraads et al., 2019) or in the current pre-adolescent study. This suggests that subcortical structures are not related to a history of stuttering in a general population.

We observed differences in white matter structures in MD but not FA. Previous literature in children and adults with stuttering reported mostly on FA differences rather than MD (Chow and Chang, 2017, Cykowski et al., 2010, Sommer et al., 2002, Watkins et al., 2008). MD has only been reported in a few studies (Kronfeld-Duenias et al., 2016a, Kronfeld-Duenias et al., 2016b, Neef et al., 2018). Kronfeld-Duenias et al. (2016b) found significantly increased MD in the left corticospinal tract and no FA differences along any of the tracts in adults who persist in stuttering compared to fluent controls. We found similar results in pre-adolescents who recovered from stuttering. They additionally found higher MD in the bilateral FAT, which partly overlaps with our bilateral SLF findings. This study speculated that increased MD and reduced FA values could stem from noisy communication (i.e., reduced synchrony) in the tract between one region to another region which could have led to excessive pruning of axons, as well as a more coherent fiber organization within the tract, which would elevate FA back to its typical range. Neef et al. (2018) also found increased MD, however not tested statistically, in all clusters with a reduced FA (e.g., tract between left SLF and IFG) in adults who persist in stuttering compared to fluent controls. This study speculated that adults who stutter exhibit a weakened connectivity of tracts along the major fiber direction, and therefore, atypical structures are insufficiently myelinated or axonal packing is reduced. This could have been the case for both recovered and persistent pre-adolescents in our study, however, we did not find this in our persistent group. Contrasting to our study, another study by Kronfeld-Duenias et al. (2016a) reported no MD difference in the language stream, which includes dorsal (SLF and FAT) and ventral tracts (ILF and uncinated fasciculus), and lower FA in the right dorsal tract in adults with stuttering. In general, while FA quantifies the fraction of diffusion that is anisotropic, MD quantifies the total diffusion within a voxel (i.e., average of the three eigenvalues). A difference in MD without a difference in FA suggests an overall difference in diffusion which is not specific to one direction of diffusivity, and thus does not translate into a difference in anisotropy (Kronfeld-Duenias et al., 2016b). Based on previous work, we expected to find higher FA in brain regions involved in speech production in pre-adolescents who recovered, but rather found higher MD. We speculate that pre-adolescents who recovered from stuttering in our study may have developed weaker microstructure (i.e., reduced connection) interconnecting frontotemporal regions and sensorimotor areas of the cortex. As this study was cross-sectional, directionality cannot be implied and these differences could have been a cause or a consequence of stuttering. These regions are known for involvement in speech motor control and auditory feedback, and could have played a role in the dysfluency of speech in pre-adolescents who eventually recovered from stuttering. Information about assisted or spontaneous recovery would be interesting for better interpretation of findings in the recovered group. Unfortunately, our study does not have suitable information on stuttering severity or the type of stuttering therapy, intensity of therapy, or outcome. Future research investigating the effectiveness of stuttering intervention should consider to include neuroimaging. This would useful in severe confounding by indication since the most severe stuttering cases get treated which would introduce bias. Therefore, randomized clinical trials which evaluate a stuttering therapy should include neuroimaging to evaluate any treatment effect on the brain. Based on our findings, persistent stuttering may be associated with gray matter deficit only, and contrary to our hypothesis, it may be not associated with white matter micro-structures. However, of important note, the group of children who persisted in stuttering was relatively small, and it is possible we were underpowered to detect a difference. Furthermore, we may have been unable to find an association if the stuttering problems in the persistent stuttering group were relatively mild, however stuttering severity was not assessed in the study and could not be examined further.

Further, in contrast to our hypothesis, it is possible that recovery from stuttering is associated with only alterations in white matter structure and not in gray matter structures. Another possibility is that recovery from stuttering in childhood could be accompanied by a dynamic neural processes. For example, structural abnormalities related to stuttering may disappear along with stuttering around the time a child experiences remission. Several examples exist of the plastic nature of the brain, including after learning, music training and motor skill practice (Bengtsson et al., 2005, Scholz et al., 2009, Zatorre et al., 2012). Importantly, a child could also experience remission of stuttering which is accompanied by functional, but not structural, reorganization in the brain. This could then manifest as a stable morphological difference in the brain. Finally, given the fact that this study included pre-adolescents only, the (micro-)structural neuroanatomy might be different in this general population relative to those found in earlier studies.

Several strengths and limitations of our study should be mentioned. Though this is the largest neuroimaging study of stuttering in pre-adolescents to date, the sample size of those who stuttered are still comparable to previous studies of stuttering (Chow and Chang, 2017, Garnett et al., 2018). Second, we used parental reports of stuttering at eight to twelve years of age, as it was not possible to assess stuttering through an in-person assessment. While a combination of parent’s reports with expert judgements remain the traditional criteria for classifying stuttering, over- or underreporting of stuttering is unlikely, as the incidence of stuttering in our study population is in line with the expected 5–10% prevalence rate (Boyle et al., 2011, Craig et al., 2002, Dworzynski et al., 2007, Månsson, 2000, Reilly et al., 2013). Third, the current study restricted analyses to a set of a priori areas based on previous literature (Beal et al., 2013, Chang et al., 2008, Garnett et al., 2018, Koenraads et al., 2019) and a widely-used neuroanatomical atlas (Desikan et al., 2006). Furthermore, these areas were relatively coarse compared to more fine-grained segmentation in these studies. However, impressive advances in the field now allow for more fine-grained characterization of brain features in stuttering and can be explored, potentially focusing on areas already identified to be related to stuttering. For example, the multimodal parcellation atlas which incorporates data from several modalities with over 300 parcellations, will be a priority in the future for studying stuttering (Glasser et al., 2016). Finally, previous studies have mostly focused on right-handed boys and the few that examined recovery and persistency in childhood stuttering used participants from predominantly small, clinic-based settings or were recruited for specific reasons (Chow and Chang, 2017, Garnett et al., 2018). In our study, the stuttering groups and the fluently speaking reference sample were both recruited from the general population. Thus, the results of this work have the potential to be more generalizable to the general population of pre-adolescents who stutter, as the sample better represents the general population in the context of both the reference sample and the stuttering phenotypes (e.g., severe stuttering phenotypes who seek medical care as well as mild stuttering phenotypes which would do not seek medical care).

However, this study is cross-sectional in nature and more work is needed to examine how stuttering is related to changes in structural cortical and subcortical brain regions over time in a longitudinal study design. Tracking the association of brain growth differences in these structures in relation to childhood stuttering over a period of time will improve insight in long term outcomes in stuttering. In addition, the etiological origins of the neurobiological features of stuttering are still unknown. It will be critical to study whether other potential etiological factors, such as genetic, psychological, and environmental in combination with neurobiological markers can predict the developmental course of stuttering alongside brain image data. Lastly, the current study only observed differences between the children who recovered from stuttering and the reference group, and no significant (though trend) differences were observed in the group who persisted in stuttering. The group of children who persisted was relatively small, was potentially underpowered, and the stuttering severity was not assessed. The group of children who recovered was also not further stratified into assisted or spontaneous recovery since proper medical information about treatment for stuttering (e.g., type, frequency, outcome) is missing. In the future, studies should aim to assess whether treatment has effect on the brains of children who develop stuttering.

In conclusion, the our findings provide evidence for structural differences in speech-relevant brain areas of pre-adolescents with persistent and recovered stuttering from a population-based study. Relatively small differences in size of effect in prefrontal gray matter are associated with persistent stuttering, and alterations in white matter tracts are apparent in those who recovered. Although comparison of the current findings with previous reports is challenging due to methodological differences, these results further strengthen the potential relevance of brain structure in developmental stuttering.

## Funding

This research project did not receive any specific grant from funding agencies in the public, commercial, or not-for-profit sectors. The general design of the Generation R Study is made possible by financial support from the Erasmus Medical Center, Rotterdam, the Erasmus University Rotterdam, ZonMw, the Netherlands Organization for Scientific Research (NWO), and the Ministry of Health, Welfare and Sport. The work of HT is supported a NWO‐VICI grant (NWO‐ZonMW: 016.VICI.170.200). RLM and neuroimaging infrastructure was supported by the Sophia Foundation (S18-20) and an Erasmus University Fellowship. Neuroimaging and neuroimaging infrastructure was also supported by the Netherlands Organization for Health Research and Development (ZonMw) TOP grant awarded to T. White (project number 91211021). Supercomputing resources were provided by the Dutch Organization for Scientific Research (NWO, SurfSara.nl).

## CRediT authorship contribution statement

**S.P.C. Koenraads:** Conceptualization, Methodology, Formal analysis, Investigation, Writing - original draft, Visualization. **M.P. van der Schroeff:** Conceptualization, Writing - original draft, Supervision, Project administration. **G. van Ingen:** Investigation, Writing - review & editing. **S. Lamballais:** Methodology, Software, Validation, Writing - review & editing. **H. Tiemeier:** Writing - review & editing. **R.J. Baatenburg de Jong:** Writing - review & editing, Supervision. **T. White:** Data curation, Writing - review & editing. **M.C. Franken:** Conceptualization, Writing - original draft, Supervision. **R.L. Muetzel:** Conceptualization, Methodology, Software, Data curation, Writing - original draft, Visualization, Supervision, Project administration.

## Declaration of Competing Interest

The authors declare that they have no known competing financial interests or personal relationships that could have appeared to influence the work reported in this paper.
