# Supplementary material for: Structural brain differences in pre-adolescents who persist in and recover from stuttering
Source: Neuroimage Clin. 2020 Jun 29;27:102334. doi: 10.1016/j.nicl.2020.102334 (PMC7341447; doi:10.1016/j.nicl.2020.102334)
Supplement: Supplementary data 1 [file mmc1.docx]

**Supplementary Tables**

Supplementary Table A.1. Demographics for all participants with structural data (n=1969)

|  | **Fluent controls**   n = 1884 | **Children who recovered from stuttering** n = 66 | **Children who persisted in stuttering** n = 19 | **p-value** |
| --- | --- | --- | --- | --- |
| **Child** |  |  |  |  |
| Age at questionnaire (years)  Age at MRI (years) (mean, SD)  Sex (boy)  Ethnicity (western)  Handedness (right)  Lingualism (monolingual) Ever had treatment for stuttering  (% yes) Ever had stutter, speech and/or language therapy (%yes) | 9.8 (0.3)  10.1 (0.6)  912 (48.4)  1454 (77.8) [n=1870]  1677 (89.3) [n=1877]  1493 (80.5) [n=1855] -  322 (17.1) | 9.8 (0.2)  10.03 (0.5) 46 (69.7)  50 (75.8)  60 (92.3) [n=65]  52 (78.8) 25 (37.9)  30 (45.5) | 9.8 (0.4)  9.9 (0.6)  15 (78.9)  13 (68.4)  15 (78.9)  14 (73.7) 14 (73.7)  15 (78.9) | > 0.05  > 0.05  0.01^a^, 1E-3^b^  > 0.05  > 0.05  > 0.05 0.01^c^  8E-4^a^, 2E-7^b^, 0.02^c^ |
| IQ at 5 years (score)  Total behavior (mean, SD)  problem score (median, IQR) | 105.0 (14.4) [n=1677]  15.9 (14.1) [n=1805] 12.0 (6.0 – 21.7) | 102.9(15.3) [n=58]  15.9 (13.9) [n=66] 13.5 (7.0 – 20.0) | 104.8 (14.8) [n=17]  25.9 (17.9) [n=18]  24.0 (10.1 – 37.2) | > 0.05  3E-3^a^, 0.01^c^ 4E-3^a^, 0.01^c^ |
| **Maternal** |  |  |  |  |
| Ethnicity (Western)  Education level (high) | 1394 (74.6) [n=1869]  1198 (67.6) [n=1772] | 48 (72.7)  45 (70.3) [n=64] | 12 (63.2)  8 (50.0) | > 0.05  > 0.05 |

All categorical variables are presented with numbers (n) and percentages (%); all continuous variables are presented as mean with standard deviation (SD).
 ^a^ persistent stuttering > fluent controls (p-value < 0.05)
^b^ recovered from stuttering > fluent controls (p-value < 0.05)
^c^ persistent stuttering > recovered stuttering (p-value < 0.05)

Supplementary Table B.1. Overview of gray and white matter morphometry differences associated with stuttering

| **Regions^#^** | **Left hemisphere** | | | **Right hemisphere** | | |
| --- | --- | --- | --- | --- | --- | --- |
|  | **Volume** | **Thickness** | **Surface area** | **Volume** | **Thickness** | **Surface area** |
| **Gray matter** |  |  |  |  |  |  |
| ***Cortical structures*** |  |  |  |  |  |  |
| Frontal Superior frontal Caudal anterior cingulate Caudal middle frontal Inferior frontal gyrus  - Pars opercularis  - Pars triangularis Lateral orbitofrontal  Insula Postcentral Precentral | P < C^+^, P < R*  -  P > C^+^  -  -  -  -  -  - | -  -  -  -  R > C^+^  -  -  -  - | -  -  -  -  -  -  -  - | -  -  -  - | -  -  -  - | -  -  -  - |
| Temporal  Transverse temporal Superior temporal Middle temporal Inferior temporal | R > C^+^ -  -  - | R > C^+^ - - P > C^+^ | - - - | - - | - - | P < C^+^, P < R^+^ P < C^+^ |
| Parietal Supramarginal Superior parietal Inferior parietal | -  -  - | P < C^+^, P < R^+^  -  - | -  -  R > C^+^ | -  - | -  - | -  - |
| ***Subcortical structures*** |  |  |  |  |  |  |
| Caudate Putamen Pallidum Thalamus | -  -  -  - |  |  | -  -  -  - |  |  |
| **White matter** |  |  |  |  |  |  |
| Corpus callosum Anterior Mid anterior Central Mid posterior Posterior | - R > C^+^  -  -  - |  |  |  |  |  |

P = persistent, R = recovered, C = fluent controls
Linear regression with model: brain region of interest = stuttering + age + gender + handedness + bilingualism + ethnicity + maternal education + intracranial volume (in volumetric outcomes only)
The number of participants varied in each analysis: P vs R (n=1902), P vs R (n= 84), R vs C (n=1949).
# regions are selected based on previous literature
* p_FDR_ <0.05
+ significant p-value <0.05 and did not survive FDR correction

Supplementary Table C.1 Linear regression analysis in gray and white matter volume, left hemisphere

|  | **Persistent vs Controls** | | | | **Recovered vs Controls** | | | | **Persistent vs Recovered** | | | |
| --- | --- | --- | --- | --- | --- | --- | --- | --- | --- | --- | --- | --- |
|  | **β** | **95% CI** | **P** | **P_FDR_** | **β** | **95% CI** | **P** | **P_FDR_** | **β** | **95% CI** | **P** | **P_FDR_** |
| **Gray matter** |  |  |  |  |  |  |  |  |  |  |  |  |
| ***Cortical structures*** |  |  |  |  |  |  |  |  |  |  |  |  |
| Frontal Superior frontal Caudal anterior cingulate Caudal middle frontal Inferior frontal gyrus   - Pars opercularis  - Pars triangularis Lateral orbitofrontal  Insula Postcentral Precentral | -1344 89 563  -303 -6 -27 242 81 218 | -2407;-280 -24;203 22;1104  -700;94 -347;333 -395;339 -87;572 -488;651 -358;796 | 0.01 0.43 0.04  0.14 0.97 0.88 0.15 0.78 0.46 | 0.16  0.92  0.32   0.48  0.97  0.97  0.48  0.97  0.92 | 116 -5 -52  -48 26 38 71 -43 -43 | -455;689 -67;56 -264;159  -251;154 -119;171 -167;245 -102;245 -352;265 -338;251 | 0.69 0.93 0.73  0.66 0.78 0.71 0.43 0.78 0.79 | 0.84 0.93 0.84  0.84 0.84 0.84 0.84 0.84 0.84 | -1825 65 522  -396 -70 -27 165 173 133 | -2999;-650 -193;325 -138;1183  -920;128 -547;407 -474;419 -221;552 -507;854 -557;824 | 3E-3 0.62 0.12  0.14 0.77 0.91 0.40 0.62 0.71 | 0.05* 0.83 0.48  0.48 0.87 0.91 0.83 0.83 0.87 |
| Temporal Transverse temporal Superior temporal Middle temporal Inferior temporal | -8 72 -29 -175 | -104;86 -600;745 -730;671 -927;576 | 0.85 0.83 0.93 0.65 | 0.97  0.97  0.97  0.97 | 50 100 200 -74 | -1;102 -2.69;470 -176;577 -498;349 | 0.05 0.59 0.29 0.72 | 0.80 0.84 0.84 0.84 | -59 101 -287 -303 | -175;56 -788;992 -1088;513 -1192;586 | 0.31 0.82 0.48 0.50 | 0.83 0.87 0.83 0.83 |
| Parietal Supramarginal Superior parietal Inferior parietal | 341 591 61 | -534;1217 -198;1382 -818;940 | 0.45 0.14 0.89 | 0.92  0.48  0.97 | -298 -301 247 | -771;173 -710;128 -261;725 | 0.22 0.17 0.31 | 0.84 0.84 0.84 | 814 828 -284 | -241;1869 -298;1955 -1370;800 | 0.13 0.15 0.61 | 0.48 0.48 0.83 |
| ***Subcortical structures*** |  |  |  |  |  |  |  |  |  |  |  |  |
| Caudate Putamen Pallidum Thalamus | 38 94 51 136 | -147;224 -22;212 -34;137 -81;355 | 0.69 0.42 0.24 0.22 | 0.85 0.65 0.55 0.55 | -14 -94 1 -101 | -115;85 -212;24 -45;48 -221;18 | 0.77 0.14 0.96 0.09 | 0.88 0.55 0.96 0.55 | 35 229 44 186 | -195;266 -58;518 -71.5;160 -108;481 | 0.13 0.12 0.45 0.22 | 0.55 0.55 0.65 0.55 |
| **White matter** |  |  |  |  |  |  |  |  |  |  |  |  |
| Corpus callosum Anterior Mid anterior Central Mid posterior Posterior | -15 24 29 -6 12 | -71;41 -40;90 -30;88 -44;31 -42;67 | 0.60 0.45 0.33 0.72 0.64 | 0.98 0.82 0.73 0.98 0.98 | 27 -37 -22 -1 1 | -2;58 -72;-2 -54;9 -22;21 -13;17 | 0.08 0.04+ 0.18 0.98 0.92 | 0.53 0.53 0.53 0.98 0.98 | -44 51 52 3 1 | -121;-9 -29;131 -16;121 -41;48 -74;78 | 0.19 0.21 0.14 0.87 0.96 | 0.53 0.53 0.53 0.98 0.53 |

Linear regression with model: brain region of interest = stuttering + age + sex + handedness + bilingualism + ethnicity + maternal education + total intracranial volume (ICV, for volumetric outcomes only)
Beta coefficients are presented in volume in mm^3^
The number of participants varied in each analysis: P vs R (n=1902), P vs R (n= 84), R vs C (n=1949).
* p_FDR_ < 0.05

Supplementary Table D.1. Linear regression analysis in gray matter thickness, left hemisphere

|  | **Persistent vs Controls** | | | | **Recovered vs Controls** | | | | **Persistent vs Recovered** | | | |
| --- | --- | --- | --- | --- | --- | --- | --- | --- | --- | --- | --- | --- |
|  | **β** | **95% CI** | **P** | **P_FDR_** | **β** | **95% CI** | **P** | **P_FDR_** | **β** | **95% CI** | **P** | **P_FDR_** |
| **Gray matter** |  |  |  |  |  |  |  |  |  |  |  |  |
| ***Cortical structures*** |  |  |  |  |  |  |  |  |  |  |  |  |
| Frontal Superior frontal Caudal anterior cingulate Caudal middle frontal Inferior frontal gyrus   - Pars opercularis  - Pars triangularis Lateral orbitofrontal  Insula Postcentral Precentral | -0.02 -0.03 0.04  -0.04 0.00 0.02 -0.03 -0.01 0.01 | -0.08;0.04 -0.13;0.08 -0.02;0.10  -0.10;0.02 -0.06;0.06 -0.05;0.08 -0.10;0.05 -0.06;0.05 -0.04;0.07 | 0.50 0.66 0.32  0.19 0.92 0.62 0.49 0.85 0.62 | 0.80 0.81 0.75  0.70 0.92 0.81 0.80 0.92 0.81 | 0.01 -0.02 -0.01  0.01 0.04 0.01 0.00 0.02 0.01 | -0.01;0.04 -0.07;-0.04 -0.04;0.03  -0.01;0.02 0.00;0.07 -0.02;0.05 -0.03;0.04 -0.01;0.05 -0.02;0.04 | 0.57 0.59 0.64  0.65 0.04 0.43 0.82 0.21 0.49 | 0.87 0.87 0.87  0.87 0.32 0.87 0.87 0.87 0.87 | -0.04 -0.01 0.03  -0.06 -0.04 -0.01 -0.05 -0.03 0.00 | -0.11;0.03 -0.13;0.12 -0.05;0.11  -0.13;0.00 -0.11;0.03 -0.09;0.07 -0.14;0.05 -0.09;0.04 -0.07;0.07 | 0.27 0.91 0.42  0.05 0.31 0.82 0.35 0.43 0.99 | 0.60 0.97 0.60  0.40 0.60 0.94 0.60 0.60 0.99 |
| Temporal Transverse temporal Superior temporal Middle temporal Inferior temporal | 0.01 0.03 0.04 0.07 | -0.08;0.09 -0.04;0.09 -0.03;0.11 0.00;0.13 | 0.92 0.43 0.33 0.05 | 0.92 0.80 0.75 0.40 | 0.05 0.01 0.00 0.01 | 0.00;0.11 -0.02;0.05 -0.04;0.04 -0.03;0.04 | 0.04 0.55 0.90 0.77 | 0.32 0.87 0.90 0.87 | -0.06 0.02 0.03 0.06 | -0.19;0.07 -0.06;0.10 -0.05;0.11 -0.02;0.14 | 0.38 0.65 0.45 0.15 | 0.60 0.80 0.60 0.60 |
| Parietal Supramarginal Superior parietal Inferior parietal | -0.06 0.03 0.04 | -0.11;-0.00 -0.02;0.08 -0.02;0.11 | 0.05 0.22 0.20 | 0.40 0.70 0.70 | -0.01 -0.01 -0.01 | -0.04;0.03 0.03;0.02 -0.05;0.02 | 0.74 0.75 0.54 | 0.87 0.87 0.87 | -0.06 0.04 0.04 | -0.11;0.00 -0.03;0.10 -0.04;0.11 | 0.05 0.25 0.33 | 0.40 0.60 0.60 |

Supplementary Table D.2. Linear regression analysis in gray matter surface area, left hemisphere

|  | **Persistent vs Controls** | | | | **Recovered vs Controls** | | | | **Persistent vs Recovered** | | | |
| --- | --- | --- | --- | --- | --- | --- | --- | --- | --- | --- | --- | --- |
|  | **β** | **95% CI** | **P** | **P_FDR_** | **β** | **95% CI** | **P** | **P_FDR_** | **β** | **95% CI** | **P** | **P_FDR_** |
| **Gray matter** |  |  |  |  |  |  |  |  |  |  |  |  |
| ***Cortical structures*** |  |  |  |  |  |  |  |  |  |  |  |  |
| Frontal Superior frontal Caudal anterior cingulate Caudal middle frontal Inferior frontal gyrus   - Pars opercularis  - Pars triangularis Lateral orbitofrontal  Insula Postcentral Precentral | -343.5 36.5 82.3  -88.5 -33.2 -47.5 80.0 -27.3 -8.6 | -758;71 -23;96 -96;260  -211;34 -130;64 -190;95 -45;205 -246;192 -233;216 | 0.11 0.23 0.37  0.16 0.51 0.52 0.21 0.81 0.94 | 0.55 0.55 0.68  0.55 0.68 0.68 0.55 0.91 0.94 | 153.7 20.8 39.9  9.6 2.8 29.3 47.2 -7.2 48 | -72;379 -11;52 -56;136  -57;76 49;55 49;107 -20;115 -126;112 74;171 | 0.18 0.21 0.42  0.87 0.92 0.46 0.17 0.91 0.44 | 0.48 0.48 0.74  0.98 0.98 0.74 0.48 0.98 0.74 | -438.9 13.1 38.3  -109.9 -26.7 -37.7 64.8 20.8 -66.4 | -955;77 -66;92 -221;297  -275;55 -148;95 -217;142 -93;223 -268;309 -33;250 | 0.10 0.75 0.77  0.19 0.67 0.68 0.42 0.89 0.68 | 0.52 0.88 0.88  0.61 0.88 0.88 0.84 0.95 0.88 |
| Temporal Transverse temporal Superior temporal Middle temporal Inferior temporal | -10.2 -62.9 -118.1 -239.1 | -41;21 -266;140 -309;73 -462;-16 | 0.53 0.55 0.23 0.04 | 0.68 0.68 0.55 0.55 | 12.3 86.3 95.7 25.4 | -4;29 -25;197 -8;199 -95;146 | 0.16 0.13 0.07 0.69 | 0.48 0.48 0.48 0.98 | -17.6 -102.2 -208.2 -256.8 | -52;17 -381;176 -452;36 -518;4.6 | 0.33 0.47 0.09 0.05 | 0.84 0.84 0.52 0.52 |
| Parietal Supramarginal Superior parietal Inferior parietal | 116.6 -33.2 -200.2 | -201;434 -376;309 -531;131 | 0.46 0.85 0.24 | 0.68 0.91 0.55 | 0.05 14.4 193.4 | -172;172 -173;201 -13;373 | 0.99 0.88 0.04 | 0.99 0.98 0.48 | 192.2 -8.4 -342.3 | -235;619 -500;484 -746;101 | 0.39 0.97 0.13 | 0.84 0.97 0.52 |

Linear regression with model: brain region of interest = stuttering + age + sex + handedness + bilingualism + ethnicity + maternal education
Beta coefficients are presented in thickness in mm, and in surface area in mm^2^
The number of participants varied in each analysis: P vs R (n=1902), P vs R (n= 84), R vs C (n=1949).

Supplementary Table E.1. White matter tracts differences associated with stuttering, in mean diffusivity (MD)

| **Regions** | **Persistent vs Fluent controls** | | | | **Recovered vs Fluent controls** | | | | **Persistent vs Recovered** | | | |
| --- | --- | --- | --- | --- | --- | --- | --- | --- | --- | --- | --- | --- |
|  | **β** | **95% CI** | **P** | **P_FDR_** | **β** | **95% CI** | **P** | **P_FDR_** | **β** | **95% CI** | **P** | **P_FDR_** |
| **Left hemisphere** |  |  |  |  |  |  |  |  |  |  |  |  |
| Superior longitudinal fasciculus Inferior longitudinal fasciculus **Forceps minor** ^o^ Forceps major ^o^ Uncinate fasciculus Inferior fronto-occipital Corticospinal tract Posterior thalamic radiation | -0.371 -0.526 -0.407  1.120 -0.437 -1.093 -0.618 -0.507 | -1.440;0.699 -1.694;0.643 -1.907;1.092 -1.963;4.202 -1.414;0.539 -2.204;0.018 -2.081;0.845 -2.260;1.245 | 0.50 0.41 0.60 0.48 0.38 0.05 0.41 0.57 | 0.71 0.71 0.71 0.71 0.71 0.71 0.71 0.71 | \| 0.848 0.613 0.248 2.119 0.288 0.645 3.175 0.711 \| \| --- \| | \| 0.292;1.404 -0.039;1.264 -0.536;1.032  0.505;3.732 -0.223;0.799  0.062;1.228  1.854;4.495 -0.217;1.640 \| \| --- \| | 3E-3 0.07 0.54 0.01 0.27 0.03 2E-6 0.13 | 0.01* 0.10 0.54 0.02* 0.32 0.05 3E-5* 0.17 | \| -1.437 -1.270 -1.027 -1.275 -0.862 -1.845 -6.478 -1.407 \| \| --- \| | \| -2.904; 0.030 -2.885; 0.345 -2.698; 0.644 -4.844; 2.295 -2.126; 0.403 -3.256;-0.435 -18.496;5.540 -4.221; 1.408 \| \| --- \| | 0.06 0.12 0.23 0.48 0.18 0.01 0.29 0.33 | 0.15 0.27 0.29 0.48 0.28 0.13 0.34 0.35 |
| **Right hemisphere** |  |  |  |  |  |  |  |  |  |  |  |  |
| Superior longitudinal fasciculus Inferior longitudinal fasciculus **Forceps minor** ^o^ Forceps major ^o^ Uncinate fasciculus Inferior fronto-occipital Corticospinal tract Posterior thalamic radiation | \| -0.530 -0.197 - - -0.362 -0.714 -0.647 -0.425 \| \| --- \| | \| -1.762;0.701 -1.680;1.285 - - -1.396;0.673 -1.949;0.521 -3.222;1.928 -2.322;1.472 \| \| --- \| | 0.40 0.80 - - 0.49 0.26 0.62 0.66 | 0.71 0.79 - - 0.71 0.71 0.71 0.71 | \| 1.018 1.307 - - 0.419 0.783 0.537 1.369 \| \| --- \| | \| 0.375;1.660 0.538;2.076 - - -0.121;0.960  0.164;1.401 -0.791;1.865  0.374;2.364 \| \| --- \| | 2E-3 1E-3 - - 0.13 0.02 0.43 0.01 | 0.01* 0.01* - - 0.17 0.04* 0.46 0.02* | \| -1.946 -1.636 - - -0.794 -1.612 -1.291 -2.137 \| \| --- \| | \| -3.572;-0.320 -3.925; 0.652 - - -2.058; 0.470 -3.075;-0.149 -2.524;-0.059 -4.934; 0.661 \| \| --- \| | 0.02 0.16 - - 0.22 0.03 0.04 0.13 | 0.13 0.28 - - 0.29 0.14 0.14 0.27 |

Linear regression with model: brain tract of interest = stuttering + age + sex + handedness + bilingualism + ethnicity + maternal education
Beta coefficients (β) are presented in mean diffusivity (MD) and have been scaled by a factor of 1000 and are reported in 10^3^ mm^2^/s
The number of participants varied in each analysis: P vs R (n=1850), P vs R (n= 81), R vs C (n=1897). ^o^ forceps minor and major: one brain region of interest, not left and right separately
* p_FDR_ < 0.05
